# Digital image analysis of Ki67 proliferation index in breast cancer using virtual dual staining on whole tissue sections: clinical validation and inter-platform agreement

**DOI:** 10.1007/s10549-018-4669-2

**Published:** 2018-01-18

**Authors:** Timco Koopman, Henk J. Buikema, Harry Hollema, Geertruida H. de Bock, Bert van der Vegt

**Affiliations:** 1 Department of Pathology and Medical Biology, University of Groningen, University Medical Center Groningen, PO Box 30001, 9700 RB Groningen, The Netherlands; 2 Department of Epidemiology, University of Groningen, University Medical Center Groningen, PO Box 30001, 9700 RB Groningen, The Netherlands

**Keywords:** Breast cancer, Ki67 proliferation index, Immunohistochemistry (IHC), Virtual dual staining, Digital image analysis (DIA), Inter-platform agreement

## Abstract

**Purpose:**

The Ki67 proliferation index is a prognostic and predictive marker in breast cancer. Manual scoring is prone to inter- and intra-observer variability. The aims of this study were to clinically validate digital image analysis (DIA) of Ki67 using virtual dual staining (VDS) on whole tissue sections and to assess inter-platform agreement between two independent DIA platforms.

**Methods:**

Serial whole tissue sections of 154 consecutive invasive breast carcinomas were stained for Ki67 and cytokeratin 8/18 with immunohistochemistry in a clinical setting. Ki67 proliferation index was determined using two independent DIA platforms, implementing VDS to identify tumor tissue. Manual Ki67 score was determined using a standardized manual counting protocol. Inter-observer agreement between manual and DIA scores and inter-platform agreement between both DIA platforms were determined and calculated using Spearman’s correlation coefficients. Correlations and agreement were assessed with scatterplots and Bland–Altman plots.

**Results:**

Spearman’s correlation coefficients were 0.94 (*p* < 0.001) for inter-observer agreement between manual counting and platform A, 0.93 (*p* < 0.001) between manual counting and platform B, and 0.96 (*p* < 0.001) for inter-platform agreement. Scatterplots and Bland–Altman plots revealed no skewness within specific data ranges. In the few cases with ≥ 10% difference between manual counting and DIA, results by both platforms were similar.

**Conclusions:**

DIA using VDS is an accurate method to determine the Ki67 proliferation index in breast cancer, as an alternative to manual scoring of whole sections in clinical practice. Inter-platform agreement between two different DIA platforms was excellent, suggesting vendor-independent clinical implementability.

**Electronic supplementary material:**

The online version of this article (10.1007/s10549-018-4669-2) contains supplementary material, which is available to authorized users.

## Introduction

Breast cancer is the most common type of cancer among women worldwide, and one of the leading causes of cancer-related death [1]. A diversity of histological and molecular parameters exists to predict prognosis and survival [2]. Immunohistochemistry for Ki67 (MKI67), a nuclear antigen which is present in all but the G0 phase of the cell cycle and therefore expressed in proliferating cells, can be used to determine tumor proliferation index [3]. Ki67 is a prognostic and predictive marker in breast cancer patients used in both clinical practice and clinical trials [4, 5]. However, Ki67 staining is subject to intra-tumoral heterogeneity and Ki67 scoring is prone to inter- and intra-observer variability, especially with ‘eyeballing’ [6–10]. Manual counting is time-consuming as at least 500–1000 cells have to be counted to achieve acceptable error rates and to correct for heterogeneity [4, 5].

Recently, digital image analysis (DIA) has emerged as a reproducible and less time-consuming alternative to manual scoring of Ki67 in breast cancer, which potentially offers a standardized diagnostic solution [4, 5, 11]. Several studies report high concordance between manual scoring and DIA [12–14]. However, these studies focus mainly on small tumor areas, either tissue microarrays (TMAs) or specific regions of interest (ROIs) within larger sections, which does not take into account intra-tumoral Ki67 heterogeneity. In clinical practice, Ki67 scoring is often performed on whole tissue sections, which is also promoted by the International Ki67 in Breast Cancer Working Group, who recommends ‘an approach that assesses the whole section’ [5]. For DIA on Ki67-stained sections, the distinction between tumor and non-tumor tissue is vital to avoid over- or underestimation of Ki67 proliferation index due to counting of non-neoplastic cells. However, manual tumor outlining in the large tissue areas of whole tumor sections is impractical, and tissue classifiers based on morphological characteristics can be relatively inaccurate [15–17]. Physical dual staining offers a possible solution, by identifying tumor with cytokeratin in addition to Ki67 on the same section, but DIA on this method is impaired by overlapping chromogens and pixel intensities of both stains [18, 19]. A novel method which circumvents this issue is virtual dual staining (VDS), in which serial sections stained with Ki67 and cytokeratin are digitally aligned [14, 15]

Studies comparing manual scoring and DIA have used different platforms by various vendors, which have unique image analysis algorithms to determine Ki67 proliferation index [12–17]. As these algorithms have different approaches to classify tissue and cellular components, inter-platform variability may be expected [15, 20]. To the best of our knowledge, all studies up to this date have implemented only one DIA platform per study and therefore have not examined inter-platform agreement.

The aims of this study were to validate DIA of Ki67 in breast carcinomas in a clinical setting using VDS on whole sections by comparing a manual whole section scoring protocol with automated scoring, and to assess inter-platform agreement between two independent DIA platforms.

## Materials and methods

Resection specimens of 154 consecutive primary invasive breast carcinomas treated in the University Medical Center Groningen (The Netherlands) between August 2015 and February 2017 were prospectively included. Patient and tumor characteristics are shown in Table 1.

**Table 1:** Patient and tumor characteristics

	All cases, *n* (%)		DIA cases^a^, *n* (%)	
Total	154	(100)	117	(100)
Gender				
Female	151	(98.1)	115	(98.3)
Male	3	(1.9)	2	(1.7)
Age (years)				
< 60	74	(48.1)	56	(47.9)
≥ 60	80	(51.9)	61	(52.1)
Mean	60.4		60.5	
Histologic type				
Ductal/no special type	132	(85.7)	101	(86.3)
Lobular	22	(14.3)	16	(13.7)
Histologic grade				
G1	37	(24.0)	28	(23.9)
G2	75	(48.7)	57	(48.7)
G3	42	(27.3)	32	(27.4)
Tumor diameter (cm)				
≤ 2	102	(66.2)	80	(68.4)
> 2 and ≤ 5	42	(27.3)	30	(25.6)
> 5	10	(6.5)	7	(6.0)
Mean	2.1		2.0	
ER				
Positive	133	(86.4)	100	(85.5)
Negative	21	(13.6)	17	(14.5)
PR				
Positive	118	(76.6)	92	(78.6)
Negative	36	(23.4)	25	(21.4)
HER2				
Positive	15	(9.7)	9	(7.7)
Negative	137	(89.0)	106	(90.6)
Equivocal	2	(1.3)	2	(1.7)

*DIA* digital image analysis*, ER* estrogen receptor, *PR* progesterone receptor*, HER2* human epidermal growth factor 2

^a^37 cases were excluded from further analysis due to misalignment of the virtual dual staining

### Immunohistochemistry

Three-micrometer serial sections were cut from formalin-fixed paraffin-embedded tumor blocks during normal clinical workflow. Adjacent sections were stained for Ki67 (CONFIRM anti-Ki-67 (30-9) rabbit monoclonal antibody, Ventana Medical Systems, Illkirch, France) and cytokeratin 8/18 (CK8/18 (B22.1 & B23.1) mouse monoclonal antibody, Ventana Medical Systems) on a Ventana BenchMark Ultra immunostainer (Ventana Medical Systems). Antibodies were pre-diluted by the manufacturer and staining was performed following the manufacturer’s protocols. Antigen retrieval times were 36 min for Ki67 and 64 min for CK8/18 (both using Cell Conditioning 1, pH 9, Ventana Medical Systems). Antibody incubation times were 28 min for Ki67 and 32 min for CK8/18. Antibody amplification was applied for CK8/18 (not for Ki67), using the Ventana Amplification Kit (Ventana Medical Systems).

### Image acquisition and DIA platforms

Digital images were acquired by scanning the glass slides in a Philips Ultra Fast Scanner 1.6 (Philips, Eindhoven, The Netherlands) with a 40× magnification lens, using a single focus layer without Z-stacking. Tissue detection with focus points was applied automatically to obtain the optimal image. Digitalized slides were stored on a centralized image server and a direct link with this server was established in both DIA platforms. The DIA platforms were Visiopharm Integrator System (VIS) platform version 6.9.0.2779 (Visiopharm, Hørsholm, Denmark) and HALO platform version 2.0.1061 (Indica Labs, Corrales, New Mexico, United States).

### Manual counting

Manual counting of Ki67 proliferation index was performed by a resident pathologist (TK), using a protocol based on the ‘whole section scoring protocol’ by the International Ki67 in Breast Carcinoma Working Group, with ROIs to represent the spectrum of staining in the whole section [5, 8]. On the digital image, three 0.500 mm^2^ ROIs were annotated within areas with high, medium, and low proliferation, respectively. If only two area types were present, two of three ROIs were selected in the area comprising the most common proliferation rate. Of the three ROIs, at least one ROI was selected centrally and at least one peripherally (i.e., the invasive edge) in the tumor. One of the ROIs was a hot spot, if present. In each ROI, 200 cells were counted in a ‘typewriter’ pattern (i.e., counting in rows within the ROI, from top to bottom, to assure a reproducible counting method) [7, 8]. Any definite brown nuclear staining was considered positive. Ki67 proliferation index representative of the whole tumor section was then calculated by dividing the number of Ki67 positive cells by the total number of counted cells (600 cells for each case).

### Digital image analysis

A training set of 20 randomly selected breast carcinoma cases obtained between January and August 2015, which were identically handled and stained but not included in the current study, was used to calibrate tissue classification by CK8/18 in VDS and nuclear classification of Ki67 in both DIA platforms. Calibration was done in close collaboration with both platform vendors, independently of each other. In both platforms, VDS was applied to digitally align corresponding Ki67- and cytokeratin-stained sections. During this process, the algorithms automatically perform distortion and rotation modifications to eliminate small differences due to tissue and section processing. Alignment was verified visually for each case, and misaligned cases were excluded from further analysis. The algorithms were then set to use the cytokeratin-stained area as the tumor classifier on the Ki67-stained section. Within the whole tissue section, the complete invasive tumor area was annotated. If present, large areas of carcinoma in situ, pre-existent epithelium, and tissue or staining artifacts were excluded. Ki67 positivity was analyzed with nuclear classification algorithms which detect nuclei by morphological form and size and classify these as positive or negative based on pixel color and intensity. In both platforms, named ‘platform A’ and ‘platform B’ henceforth, Ki67 proliferation index was calculated by dividing the number of Ki67 positive cells by the total number of positive and negative cells within the area classified as tumor by VDS. In cases with a ≥ 10% Ki67 difference by DIA versus manual counting and intra-tumoral Ki67 heterogeneity, additional DIA was performed on the manually counted ROIs only, to evaluate representativeness of these ROIs for the whole tumor.

### Statistical analysis

Spearman’s correlation coefficients were calculated for inter-observer agreement between Ki67 proliferation index by manual counting and by one of the two DIA platforms, as well as for inter-platform agreement. Scatterplots and Bland–Altman plots were created to assess inter-observer and inter-platform correlation and agreement in relation to data ranges. Plots were created and statistical analysis was performed using IBM SPSS Statistics for Windows version 23.0.0.3 (SPSS, Chicago, Illinois, United States). All testing was two sided. Values of *p* < 0.05 were considered significant.

## Results

Of the 154 cases included, VDS failed in 37 cases (24%) because of alignment issues due to relative folding or twisting of tissue, or because sections were not properly cut in serial order. VDS alignment was not influenced by CK8/18 staining or tumor size. Of these 37 cases, 32 were misaligned in both DIA platforms, 3 cases were misaligned in only one platform (as the other platform’s algorithm was able to correct the relative twisting), and 2 cases could not be aligned by one platform as the stains were mirrored. Therefore, further analysis was performed on 117 cases.

### Correlation of manual counting and DIA

**Fig. 1:**
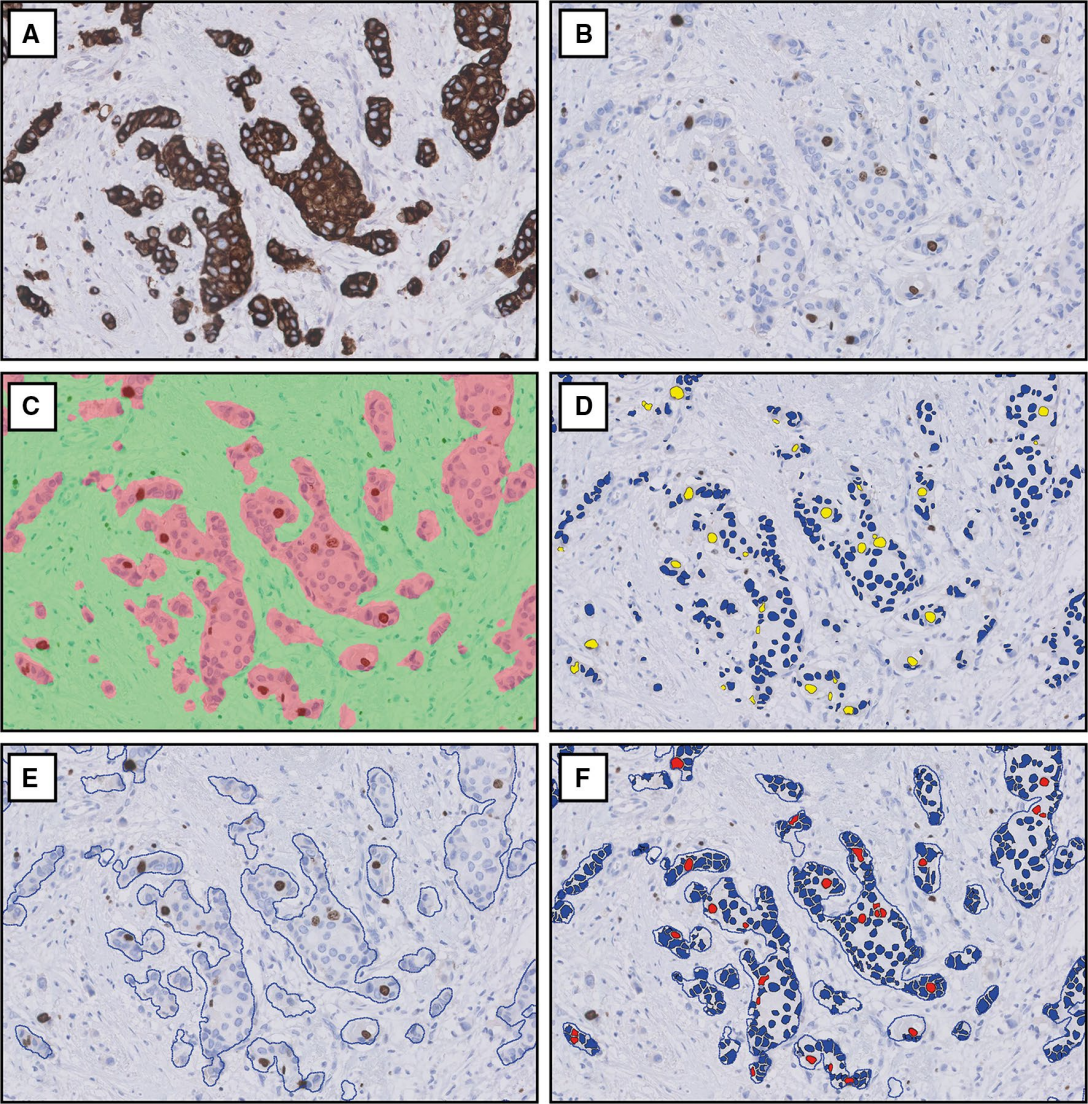
Digital image analysis of Ki67 with virtual dual staining. Corresponding cytokeratin (**a**) and Ki67 (**b**) stains are virtually aligned and Ki67 nuclear classification is determined among the cells in the area classified as tumor, shown in platform A (**c**, **d**) and platform B (**e**, **f**). Images at 200× magnification

**Fig. 2:**
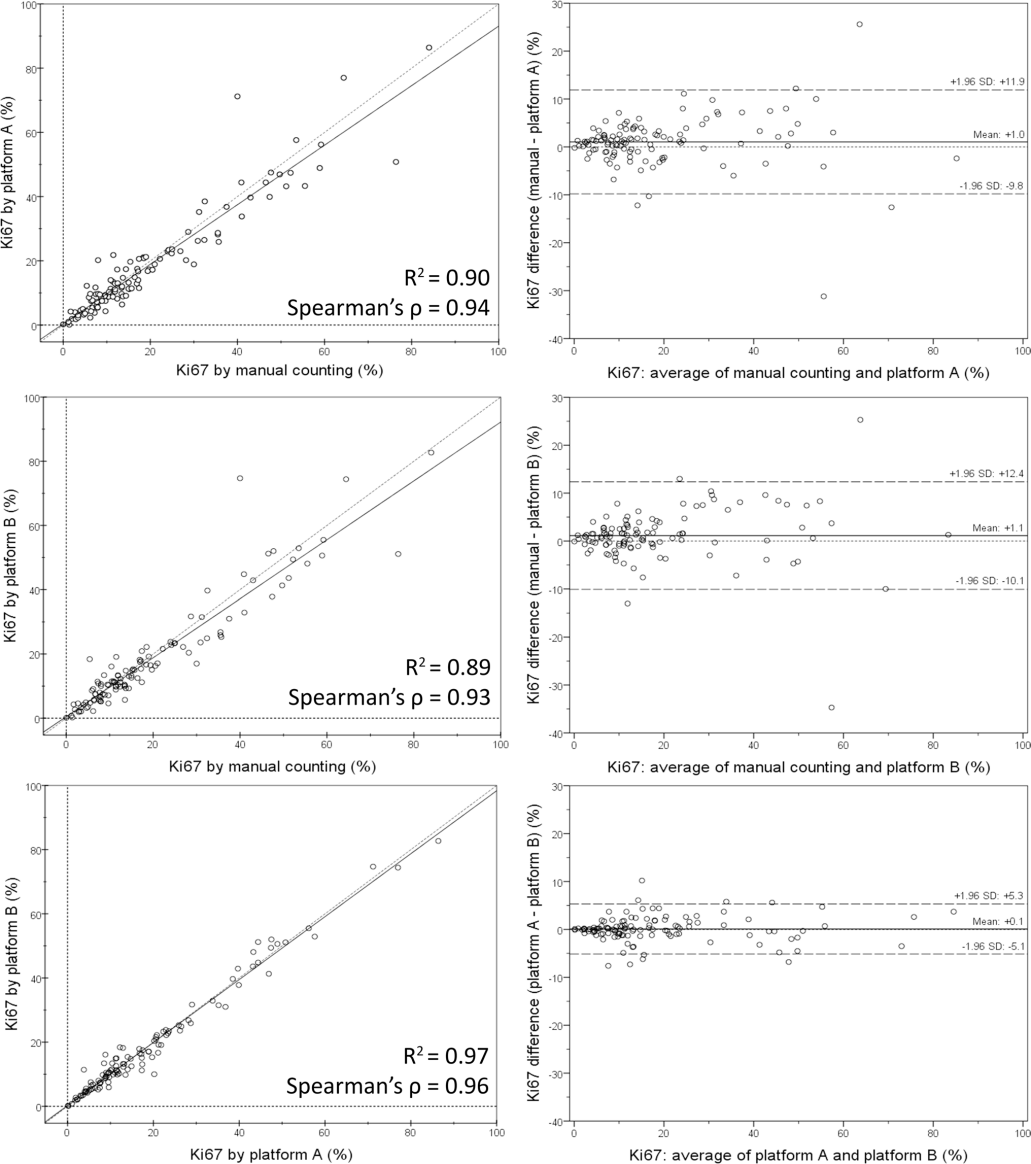
Scatterplots with correlation coefficients (left) and Bland–Altman plots of agreement (right) between whole tumor Ki67 proliferation index by manual counting versus platform A (upper row), manual counting versus platform B (middle row), and platform A versus platform B (lower row)

Manual and digital cell count profile and Ki67 proliferation index are displayed in Table 2. DIA is illustrated in Fig. 1. Ki67 scores were slightly higher by manual counting than by DIA; mean 19.5% versus 18.3–18.4% and median 13.5% versus 12.2–12.6%. Scatterplots and Bland–Altman plots of manual counting compared to both DIA platforms as well as between platforms are displayed in Fig. 2. There was no skewness within specific data ranges. Correlation for inter-observer agreement between manual counting and DIA was high: Spearman’s correlation coefficients were 0.94 (*p* < 0.001) for manual counting compared to platform A and 0.93 (*p* < 0.001) for manual counting compared to platform B. Correlation for inter-platform agreement between platform A and platform B was even higher, with a Spearman’s correlation coefficient of 0.96 (*p* < 0.001).

**Table 2:** Cell count profile and Ki67 proliferation indexes by manual counting and digital image analysis

	Mean	Min	Q1	Q2 (median)	Q3	Max
Cells counted						
Manual	600	600	600	600	600	600
Platform A	189,086	3853	53,524	131,043	281,928	715,525
Platform B	206,154	6010	63,632	154,386	299,081	889,638
Ki67, %						
Manual	19.5	0.0	7.8	13.5	26.0	84.0
Platform A	18.4	0.1	7.5	12.2	30.0	86.4
Platform B	18.3	0.1	7.5	12.6	23.1	82.7

### Cases with ≥ 10% Ki67 difference

**Fig. 3:**
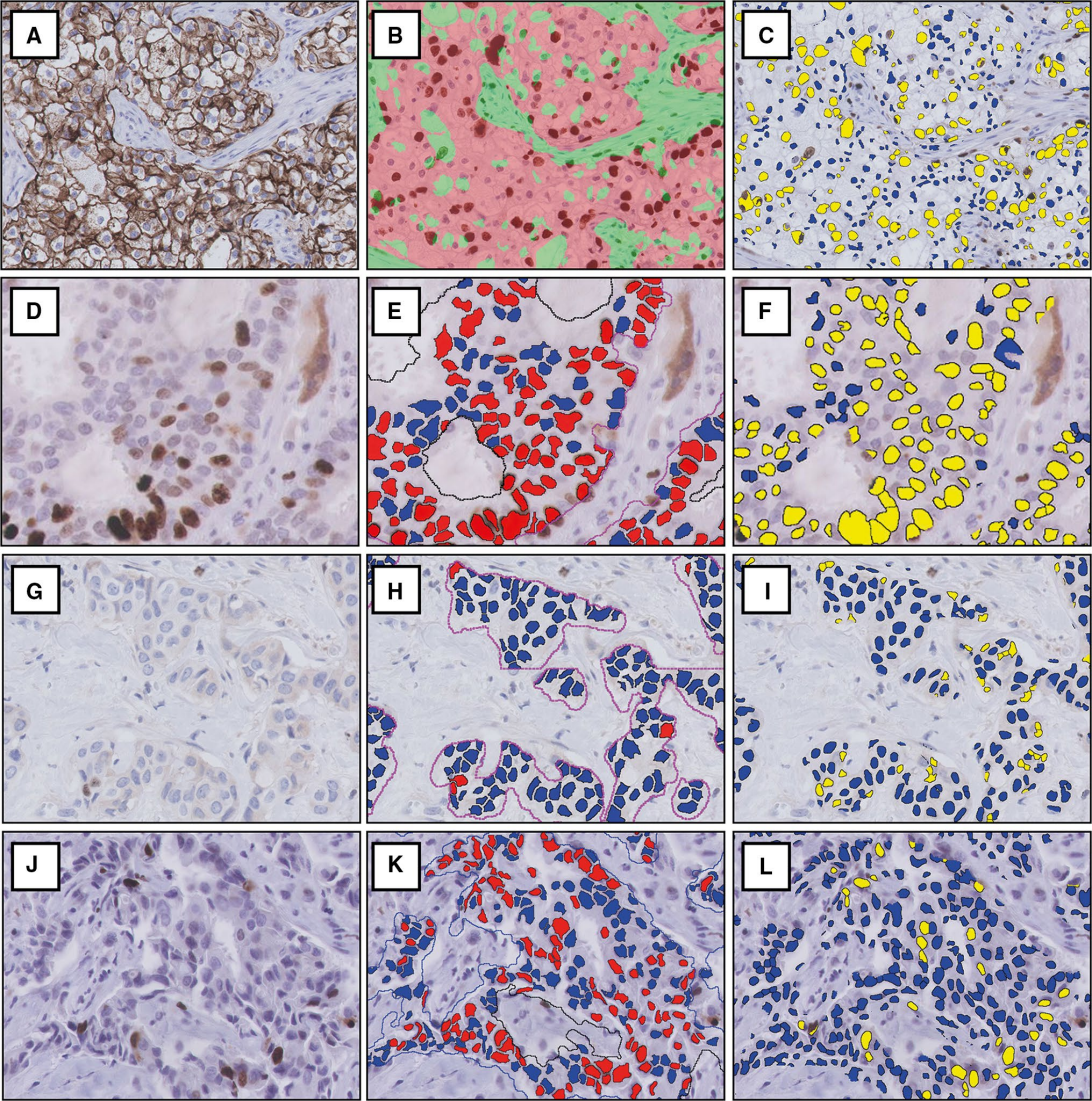
Cases with ≥ 10% difference in Ki67 proliferation index between digital image analysis and manual counting due to tumor morphology or staining artifacts. One case had clear cell morphology, causing erroneous tumor classification (**a**–**c**). Two cases had nuclear overlap and cell clustering (**d**), causing misclassification of Ki67 both by platform A (**e**) and platform B (**f**). In one case, artifactual cytoplasmic Ki67 staining (**g**) was correctly handled by platform A (**h**) but was classified as positive nuclear staining by platform B (**i**). One case with hematoxylin overstaining (**j**) led to false-positive classification of nuclei by platform A (**k**) but not by platform B (**l**). Images at 200× magnification

Ten of all 117 cases (8.5%) showed a difference in Ki67 proliferation index of ≥ 10% by DIA compared to manual counting, as shown in Table 3 and illustrated in Fig. 3. Only 2 cases had differences of > 13%. In 5 of the 10 cases, differences between manual counting and DIA were due to intra-tumoral Ki67 heterogeneity. When DIA was done on the manually counted ROIs only (instead of the whole tumor), differences were well below 10%. In the other 5 cases, the difference was due to tumor morphology or staining artifacts. Interestingly, differences between both DIA platforms were < 5% in the majority of cases (8 out of 10). Only one case had a ≥ 10% (10.2%) difference between platforms, due to hematoxylin overstaining which led to positive classification of Ki67 negative cells in platform A but not in platform B. In another case, artifactual cytoplasmic staining was erroneously recognized as positive nuclear staining by platform B but not by platform A (6.2% difference).

**Table 3:** Cases with ≥ 10% difference of Ki67 proliferation index by manual counting and DIA (total *n* = 117)

Case	Manual Ki67 %	Platform A Ki67 % (difference)^a^	Platform B Ki67 % (difference)^a^	Inter-platform difference	Reason of the difference	ROI analysis^b^, Platform A	Ki67% (difference) Platform B
1	5.4	12.2 (6.8)	18.4 (**13.0**)	6.2	Cytoplasmic Ki67 staining artifacts	–	–
2	8.0	20.2 (**12.2**)	10.0 (2.0)	10.2^c^	Nuclear hematoxylin overstaining	–	–
3	11.5	21.8 (**10.3**)	19.1 (7.6)	2.7	Ki67 heterogeneity	17.7 (6.2)	15.5 (4.0)
4	30.0	18.9 (**11.1**)	17.0 (**13.0**)	1.9	Ki67 heterogeneity	30.7 (0.7)	31.1 (1.1)
5	35.7	25.9 (9.8)	25.3 (**10.4**)	0.6	Ki67 heterogeneity	29.9 (5.8)	29.8 (5.9)
6	40.0	71.2 (**31.2**)	74.7 (**34.7**)	3.5	Cell clustering and nuclear overlap	–	–
7	55.5	43.3 (**12.2**)	48.1 (7.4)	4.8	Cell clustering and nuclear overlap	–	–
8	58.9	48.9 (**10.0**)	50.6 (8.3)	1.7	Ki67 heterogeneity	53.2 (5.7)	53.5 (5.4)
9	64.4	77.0 (**12.6**)	74.4 (**10.0**)	2.6	Ki67 heterogeneity	70.0 (5.6)	67.1 (2.7)
10	76.4	50.8 (**25.6**)	51.1 (**25.3**)	0.3	Clear cell morphology of the tumor	–	–

*DIA* digital image analysis, *ROI* region of interest

^a^Difference with manual Ki67 score, ≥ 10% differences highlighted in **bold**

^b^Difference with manual Ki67 score when DIA was performed on the manually counted ROIs instead of on the whole tumor, in cases with Ki67 heterogeneity

^c^Only this single case had a ≥ 10% difference between platform A and platform B

### Clinical context

The clinically relevant Ki67 cut-off is 20%, as defined by the St. Gallen criteria [21]. When this cut-off was applied in our study, discordance of tumor subtype classification due to Ki67 score by DIA versus manual counting occurred in 4 cases (3.4%) with platform A and 2 cases (1.7%) with platform B. Of these cases, one was among the cases with ≥ 10% difference discussed previously. All of the remaining cases were just above or just below the 20% cut-off with differences of 3.9% at most, illustrating a small margin of error (results by both platforms were similar). The degree of Ki67 differences between different counting methods in cases with Ki67 between 15 and 25% (near the 20% cut-off) is displayed in Supplementary Table 1. Clinicopathological characteristics of these cases were similar to those of the total study population (Supplementary Table 2).

## Discussion

The aims of this study were to clinically validate DIA of Ki67 using VDS on whole tissue breast carcinoma sections, and to assess inter-platform agreement between two independent DIA platforms. We found high inter-observer agreement between manual counting and DIA, and even higher inter-platform agreement.

Correlations in studies comparing manual scoring with DIA vary between 0.89 and 0.97 [12–14]. In the current study, Spearman’s correlation coefficients were 0.94 (*p* < 0.001) and 0.93 (*p* < 0.001) between manual counting versus platform A and platform B, respectively, which is in line with these studies. Only one study implemented VDS, with an intraclass correlation coefficient of 0.97 between manual counting and DIA [14]. However, that study used TMAs and thereby preselected smaller areas of the whole tumor. In clinical practice, Ki67 is often scored on whole sections, as is recommended by the International Ki67 Working Group [5]. In our study, a manual counting protocol based on the ‘whole section scoring protocol’ by this Working Group was highly concordant with DIA on whole sections. As such, we have confirmed that VDS is an accurate method to perform DIA of Ki67 on whole sections and can be used in clinical practice.

Of the initial 154 cases included in our study, a large number (37 cases; 24%) was excluded due to VDS failure, which occurred in both platforms. For successful VDS alignment, the Ki67- and cytokeratin-stained sections must be identical and accurate serial sectioning is essential. Additionally, folding or twisting of one of the sections can cause VDS misalignment. As such, it is crucial that laboratory technicians responsible for the preparation of the slides are properly instructed and trained. For this study, laboratory technicians did not receive specific instructions on the necessity of careful stretching and serial sectioning, which could be the cause of the large number of misaligned cases. For clinical implementation of VDS, we therefore recommend specific instruction and training courses for laboratory technicians on the effects of inaccurately cut and mounted sections.

Inter-platform variability between different DIA platforms may be expected as tissue morphology, cellular features, and staining patterns are handled differently depending on the platform’s algorithm [15, 20]. In clinical practice, this could lead to inconsistency of Ki67 scores when different platforms are used to perform DIA. To the best of our knowledge, the current study is the first to address inter-platform agreement on one set of tumors. Inter-platform agreement was very high, with a Spearman’s correlation coefficient of 0.96. This shows that DIA is reproducible among different platforms, and therefore a clinical pathology laboratory is not bound to a specific DIA platform or vendor, as long as the algorithm is calibrated and validated in close collaboration with the platform vendor.

In 5 cases, there was a ≥ 10% difference in Ki67 proliferation index between DIA and manual counting due to tumor morphology or staining artifacts. Results by both platforms were similar in these cases, illustrating that both platforms handle troublesome cases in a similar way. We recommend that after analysis, a quick visual check of the results by a clinical pathologist should always be performed.

Intra-tumoral heterogeneity is a known occurrence in Ki67 stains [4, 16, 22, 23]. The manual counting protocol used in our study compensated for heterogeneity in most cases, yet there were 5 cases with a ≥ 10% difference due to heterogeneity. In these cases, we performed additional DIA on the manually counted ROIs (instead of the whole tumor). In that analysis, differences became well below 10%, showing that the manually selected ROIs were inadequately representative for the whole tumor in these cases (Table 3).

In a clinical context, Ki67 can be used in the distinction of intrinsic tumor subtypes (luminal A or luminal B). Previously, DIA of the concerning surrogate biomarkers (ER, PR, HER2 and Ki67) was shown to be prognostic superior to manual scoring [15]. According to the St. Gallen criteria, the clinically relevant Ki67 cut-off is 20% [21]. When this cut-off was applied for Ki67 by DIA versus manual counting in our study, there was discordance of tumor subtype classification in only a few cases. Additionally, the difference between counting methods (Supplementary Table 1) was < 5% in the majority of all DIA cases as well as in Ki67 15–25% subgroups (near the 20% cut-off). However, even a small discrepancy can make the difference between subtyping in cases near the 20% cut-off. Whether manual counting or DIA should be the ‘gold standard’ in these cases is subject to debate; most studies have correlated clinical significance with manual counting, but others have shown that DIA is prognostically stronger [5, 11, 15]. With regard to the St. Gallen criteria, Ki67 is only of importance in tumors which are ER positive, PR positive, and HER2 negative, especially in low-grade tumors of small size [21]. However, clinicopathologic characteristics of cases near the 20% cut-off were similar to that of our total study population (Supplementary Table 2), with possibly a slightly higher ER-positivity and HER2-positivity rates. As such, no specific clinicopathological characteristic is predictive of this Ki67 subgroup, though the clinical relevance of Ki67 in ER-negative, PR-negative, and/or HER2-positive tumors could be limited.

Calibration and validation are vital to the success of DIA [9]. Calibration can be challenging, and it is important to realize that the image analysis algorithms of both platforms used in our study were calibrated on our laboratory stains and scans, in close collaboration with the platform vendors. This collaboration is important, as the pathologist has the clinical expertise, whereas the platform vendor has the technical expertise. Differences in protocols and equipment among laboratories but also within one laboratory necessitate proper and continuous calibration and validation, as differences in staining methodology and materials can lead to variable texture and color nuances which can influence DIA algorithms [14]. Further studies could investigate the performance of DIA with regard to inter-laboratory variability on identical sets of tumors. Additionally, inter-platform agreement between other platforms than the two included in this study should be investigated.

A last point of interest is the cost of DIA. Initially, DIA would seem expensive, as it requires a scanner for digitalization of the images, DIA software, and a technician to carry out the analysis. However, an increasing amount of modern pathology laboratories are incorporating digital pathology in their diagnostic workflow [24]. In addition to being more reproducible, DIA can replace time-consuming manual counting of Ki67, saving pathologists time.

In conclusion, we have shown that DIA using VDS is an accurate method to determine Ki67 proliferation index on whole sections of invasive breast carcinomas. For clinical implementation, proper training of laboratory technicians responsible for the section preparation is crucial to prevent failure of VDS alignment. DIA of Ki67 offers an objective alternative to manual Ki67 counting and has high inter-platform agreement, suggesting that it is clinically implementable independent of a specific platform vendor.

## Electronic supplementary material

Below is the link to the electronic supplementary material.

Supplementary material 1 (DOCX 16 kb)

Supplementary material 2 (DOCX 17 kb)

## Abbreviations

DIA: Digital image analysis
IHC: Immunohistochemistry
ROI: Region of interest
TMA: Tissue microarray
VDS: Virtual dual staining

## References

[1] American Cancer Society (2015). Global cancer facts & figures.

[2] Lakhani SR, Ellis IO, Schnitt SJ, Tan PH, van de Vijver MJ (2012). WHO classification of tumours of the breast.

[3] Gerdes J, Lemke H, Baisch H, Wacker HH, Schwab U, Stein H (1984). Cell cycle analysis of a cell proliferation-associated human nuclear antigen defined by the monoclonal antibody Ki-67. J Immunol.

[4] Denkert C, Budczies J, von Minckwitz G, Wienert S, Loibl S, Klauschen F (2015). Strategies for developing Ki67 as a useful biomarker in breast cancer. Breast.

[5] Dowsett M, Nielsen TO, A’Hern R (2011). International Ki67 in breast Cancer working group. Assessment of Ki67 in breast cancer: recommendations from the international Ki67 in breast Cancer working group. J Natl Cancer Inst.

[6] Polley MY, Leung SC, McShane LM (2013). An international Ki67 reproducibility study. J Natl Cancer Inst.

[7] Polley MY, Leung SC, Gao D (2015). An international study to increase concordance in Ki67 scoring. Mod Pathol.

[8] Leung SCY, Nielsen TO, Zabaglo L (2016). Analytical validation of a standardized scoring protocol for Ki67: phase 3 of an international multicenter collaboration. NPJ Breast Cancer.

[9] Laurinavicius A, Plancoulaine B, Laurinaviciene A (2014). A methodology to ensure and improve accuracy of Ki67 labelling index estimation by automated digital image analysis in breast cancer tissue. Breast Cancer Res.

[10] Varga Z, Diebold J, Dommann-Scherrer C (2012). How reliable is Ki-67 immunohistochemistry in grade 2 breast carcinomas? A QA study of the Swiss Working Group of Breast- and Gynecopathologists. PLoS ONE.

[11] Gudlaugsson E, Skaland I, Janssen EA (2012). Comparison of the effect of different techniques for measurement of Ki67 proliferation on reproducibility and prognosis prediction accuracy in breast cancer. Histopathology.

[12] Klauschen F, Wienert S, Schmitt W (2015). Standardized Ki67 diagnostics using automated scoring—clinical validation in the GeparTrio breast cancer study. Clin Cancer Res.

[13] Zhong F, Bi R, Yu B, Yang F, Yang W, Shui R (2016). A comparison of visual assessment and automated digital image analysis of Ki67 labeling index in breast cancer. PLoS ONE.

[14] Roge R, Riber-Hansen R, Nielsen S, Vyberg M (2016). Proliferation assessment in breast carcinomas using digital image analysis based on virtual Ki67/cytokeratin double staining. Breast Cancer Res Treat.

[15] Stalhammar G, Martinez NF, Lippert M (2016). Digital image analysis outperforms manual biomarker assessment in breast cancer. Mod Pathol.

[16] Christgen M, von Ahsen S, Christgen H, Langer F, Kreipe H (2015). The region-of-interest size impacts on Ki67 quantification by computer-assisted image analysis in breast cancer. Hum Pathol.

[17] Fasanella S, Leonardi E, Cantaloni C (2011). Proliferative activity in human breast cancer: Ki-67 automated evaluation and the influence of different Ki-67 equivalent antibodies. Diagn Pathol.

[18] Nielsen PS, Riber-Hansen R, Schimdt H, Steiniche T (2016). Automated quantification of proliferation with automated hot-spot selection in phosphohistone H3/MART1 dual-stained stage I/II melanoma. Diagn Pathol.

[19] Nielsen PS, Riber-Hansen R, Raundahl J, Steiniche T (2012). Automated quantification of MART1-verified Ki67 indices by digital image analysis in melanocytic lesions. Arch Pathol Lab Med.

[20] Kårsnäs A, Strand R, Doré J, Ebstrup T, Lippert M, Bjerrum K (2015). A histopathological tool for quantification of biomarkers with sub-cellular resolution. Comput Methods Biomech Biomed Eng Imaging Vis.

[21] Goldhirsch A, Winer EP, Coates AS (2013). Personalizing the treatment of women with early breast cancer: highlights of the St Gallen International Expert Consensus on the Primary Therapy of Early Breast Cancer 2013. Ann Oncol.

[22] Plancoulaine B, Laurinaviciene A, Herlin P (2015). A methodology for comprehensive breast cancer Ki67 labeling index with intra-tumor heterogeneity appraisal based on hexagonal tiling of digital image analysis data. Virch Arch.

[23] Laurinavicius A, Plancoulaine B, Rasmusson A (2016). Bimodality of intratumor Ki67 expression is an independent prognostic factor of overall survival in patients with invasive breast carcinoma. Virch Arch.

[24] Madabhushi A, Lee G (2016). Image analysis and machine learning in digital pathology: challenges and opportunities. Med Image Anal.

[25] FMWV code of conduct for health research (2011) https://www.federa.org/sites/default/files/bijlagen/coreon/code_of_conduct_for_medical_research_1.pdf. Accessed 17 May 2017

