# Supplementary material for: Digital image analysis of Ki67 proliferation index in breast cancer using virtual dual staining on whole tissue sections: clinical validation and inter-platform agreement
Source: Breast Cancer Res Treat. 2018 Jan 18;169(1):33–42. doi: 10.1007/s10549-018-4669-2 (PMC5882622; doi:10.1007/s10549-018-4669-2)
Supplement: Supplementary file 1 — Supplementary material 1 (DOCX 16 kb) [file 10549_2018_4669_MOESM1_ESM.docx]

| **Supplementary Table 1** Degree of Ki67 differences between different counting methods, in all DIA cases and in cases with Ki67 between 15-25% (i.e. near the 20% cutoff by the St. Gallen criteria [21]) | | | | |
| --- | --- | --- | --- | --- |
|  | **All DIA cases** | **Ki67 15-25%**  **(manual counting)** | **Ki67 15-25%  (platform A)** | **Ki67 15-25%  (platform B)** |
| *Total* | 117 | 24 | 23 | 27 |
|  |  |  |  |  |
| *Ki67, manual vs. platform A* |  |  |  |  |
| <5% difference | 95 (81.2%) | 23 (95.8%) | 19 (82.6%) | N/A |
| 5-10% difference | 14 (12.0%) | 1 (4.2%) | 1 (4.3%) | N/A |
| >10% difference | 8 (6.8%) | 0 (0%) | 3 (13.0%) | N/A |
|  |  |  |  |  |
| *Ki67, manual vs. platform B* |  |  |  |  |
| <5% difference | 92 (78.6%) | 23 (95.8%) | N/A | 20 (74.1%) |
| 5-10% difference | 19 (16.2%) | 1 (4.2%) | N/A | 5 (18.5%) |
| >10% difference | 6 (5.1%) | 0 (0%) | N/A | 2 (7.4%) |
|  |  |  |  |  |
| *Ki67, platform A vs. B* |  |  |  |  |
| <5% difference | 108 (92.3%) | N/A | 22 (95.7%) | 24 (88.9%) |
| 5-10% difference | 8 (6.8%) | N/A | 0 (0%) | 3 (11.1%) |
| >10% difference | 1 (0.9%) | N/A | 1 (4.3%) | 0 (0%) |
| *DIA, digital image analysis; N/A, not applicable* | | | | |
