# Supplementary material for: Digital image analysis of Ki67 proliferation index in breast cancer using virtual dual staining on whole tissue sections: clinical validation and inter-platform agreement
Source: Breast Cancer Res Treat. 2018 Jan 18;169(1):33–42. doi: 10.1007/s10549-018-4669-2 (PMC5882622; doi:10.1007/s10549-018-4669-2)
Supplement: Supplementary file 2 — Supplementary material 2 (DOCX 17 kb) [file 10549_2018_4669_MOESM2_ESM.docx]

| **Supplementary Table 2** Clinicopathological characteristics of cases with a Ki67 proliferation index between 15-25%, compared to the total study population | | | | |
| --- | --- | --- | --- | --- |
|  | **All cases** | **Ki67 15-25% (manual counting)** | **Ki67 15-25% (platform A)** | **Ki67 15-25% (platform B)** |
| *Total* | 154 | 24 | 23 | 27 |
|  |  |  |  |  |
| *Histologic type* |  |  |  |  |
| Ductal / NST | 132 (85.7%) | 19 (79.2%) | 20 (87.0%) | 23 (85.2%) |
| Lobular | 22 (14.3%) | 5 (20.8%) | 3 (13.0%) | 4 (14.8%) |
|  |  |  |  |  |
| *Histologic grade* |  |  |  |  |
| Grade 1 | 37 (24.0%) | 4 (16.7%) | 3 (13.0%) | 5 (18.5%) |
| Grade 2 | 75 (48.7%) | 15 (62.5%) | 12 (52.2%) | 14 (51.9%) |
| Grade 3 | 42 (27.3%) | 5 (20.8%) | 8 (34.8%) | 8 (29.6%) |
|  |  |  |  |  |
| *Tumor diameter (cm)* |  |  | | |
| ≤2 cm | 102 (66.2%) | 13 (54.2%) | 17 (73.9%) | 17 (63.0%) |
| 2-5 cm | 42 (27.3%) | 10 (41.7%) | 6 (26.1%) | 10 (37.0%) |
| >5 cm | 10 (6.5%) | 1 (4.2%) | 0 (0%) | 0 (0%) |
|  |  |  |  |  |
| *ER* |  |  |  |  |
| Positive | 133 (86.4%) | 24 (100%) | 21 (91.3%) | 27 (100%) |
| Negative | 21 (13.6%) | 0 (0%) | 2 (8.7%) | 0 (0%) |
|  |  |  |  |  |
| *PR* |  |  |  |  |
| Positive | 118 (76.6%) | 21 (87.5%) | 19 (82.6%) | 23 (85.2%) |
| Negative | 36 (23.4%) | 3 (12.5%) | 4 (17.4%) | 4 (14.8%) |
|  |  |  |  |  |
| *HER2* |  |  |  |  |
| Positive | 15 (9.7%) | 5 (20.8%) | 7 (30.4%) | 6 (22.2%) |
| Negative  Equivocal | 137 (89.0%)  2 (1.3%) | 19 (79.2%)  0 (0%) | 16 (69.6%)  0 (0%) | 21 (77.8%)  0 (0%) |
| *DIA, digital image analysis; NST, no special type; ER, estrogen receptor; PR, progesterone receptor; HER2, human epidermal growth factor 2* | | | | |
